# Structural and RNAi characterization of the German cockroach lipophorin receptor, and the evolutionary relationships of lipoprotein receptors

**DOI:** 10.1186/1471-2199-8-53

**Published:** 2007-06-22

**Authors:** Laura Ciudad, Xavier Bellés, Maria-Dolors Piulachs

**Affiliations:** 1 Department of Physiology and Molecular Biodiversity. Institut de Biologia Molecular de Barcelona, CSIC, Jordi Girona 18, 08034 Barcelona, Spain

## Abstract

**Background:**

Lipophorin receptors (LpRs) have been described in a number of insects, but functional studies have been reported only in locusts and mosquitoes. The aim of the present work was to characterize the LpR of the cockroach *Blattella germanica*, not only molecularly but also functionally using RNAi techniques, and to place LpRs in a phylogenetical context among lipoprotein receptors.

**Results:**

We cloned a putative LpR from *B. germanica *(BgLpR) using RT-PCR methods. Two isoforms of BgLpR that differ from each other by an insertion/deletion of 24 amino acids were obtained from the fat body and the ovary. A phylogenetical analysis of lipoprotein receptors showed that BgLpR grouped with other sequences annotated as LpR in a cluster placed as a sister group of vertebrate low density lipoprotein receptors (LDLR) + lipoprotein receptor-related proteins 8 (LPR8) + vitellogenin receptors (VgR) + very low density lipoprotein receptors (VLDLR). The two BgLpR isoforms are expressed in different adult female tissues (fat body, ovary, brain, midgut, muscle) and in embryos. In ovaries and fat body, the two isoforms are similarly expressed during the first gonadotrophic cycle. mRNA levels in the fat body increase in parallel to vitellogenesis, whereas they decrease in the ovaries. BgLpR protein levels increase in parallel to vitellogenesis in both organs. Treatment with juvenile hormone increases BgLpR protein. RNAi experiments show that females with lower BgLpR expression have less lipophorin in the growing oocytes with respect to controls.

**Conclusion:**

The two isoforms of BgLpR are structurally similar to other LpRs. Phylogenetical analyses show that LpRs and the group formed by vertebrate LDLR + LPR8 + VgR + VLDLR, diverged from a common ancestor and diversified in parallel. The different expression patterns in the fat body and the ovary, comparing mRNA and protein, indicate that the corresponding mechanisms regulating BgLpR expression are different. Experiments with JH III suggest that such a hormone regulates the expression of BgLpR posttranscriptionally. RNAi experiments indicate that BgLpR is a functional lipophorin receptor.

## Background

Lipophorins are insect proteins mainly involved in lipid transport, acting as a shuttle of lipids between different tissues[1-3]. However, lipophorin has additionally been related to other functions, for example, it plays a role as juvenile hormone (JH) binding protein in certain species, which suggests that it may contribute to the regulation of JH titers in the hemolymph [4,5]. During vitellogenesis, lipophorin transports lipids and other yolk precursors from the fat body to the ovaries and, in some species, lipophorin itself takes part of the egg yolk platelets [6]. Lipophorin incorporates into cells through receptor mediated endocytosis [7], and the first lipophorin receptor (LpR) was molecularly and functionally characterized in the locust, *Locusta migratoria *by Van der Horst's group [8]. Thereafter, cloning and sequencing of LpRs has been reported for several insect species, such as the yellow fever mosquito *Aedes aegypti *[9,10], the wax moth *Galleria mellonella *[11] and the silkworm *Bombyx mori *[12]. In *A. aegypti*, two splice variants of LpR specific to the fat body (LpRfb) and the ovary (LpRov) have been characterized by Raikhel's group [9,10], and LpRfb (and lipophorin itself) have shown to increase after immune challenge, which suggests that lipid metabolism is involved in the mosquito immune response [13].

In the German cockroach, *Blattella germanica*, lipophorin is a high density lipoprotein with a molecular weight of 670 kDa, composed of two apolipoprotein subunits of 212 (ApoLp I) and 80 kDa (ApoLp II) [4]. As stated above, the main role of lipophorins is transporting lipids through the hemolymph, and cockroach lipophorins have a substantial lipid fraction. In *Periplaneta americana*, 50% of lipophorin mass are lipids [14], and in *B. germanica *the lipid fraction represents 51% [4]. The occurrence of lipophorin in ovaries of *B. germanica *was reported by Kunkel and Pan in 1976 [15], and specific estimations of ovarian lipophorin contents were later published by Schal and coworkers [4,16], who additionally studied the role of lipophorin as hydrocarbon carrier from the hemolymph to the oocyte.

The present work aimed to characterize the LpR of *B. germanica *(BgLpR) in the fat body as well as in the ovary, not only regarding the receptor structure and organization, but also studying the function in the context of vitellogenesis. From a structural point of view, the obtained sequence of BgLpR was characterized in terms of organization and comparison with other LpRs. In addition, a phylogenetical analysis of lipoprotein receptors led to place LpRs in an evolutionary context. Furthermore, we determined the expression patterns of BgLpR in the fat body and the ovary during vitellogenesis, we studied the influence of JH on BgLpR expression, and we demonstrated the function of BgLpR as receptor mediating lipophorin incorporation into growing oocytes, following RNA interference (RNAi) experimental approaches.

## Results

### Primary structure and organization of BgLpR

**Figure 1:**
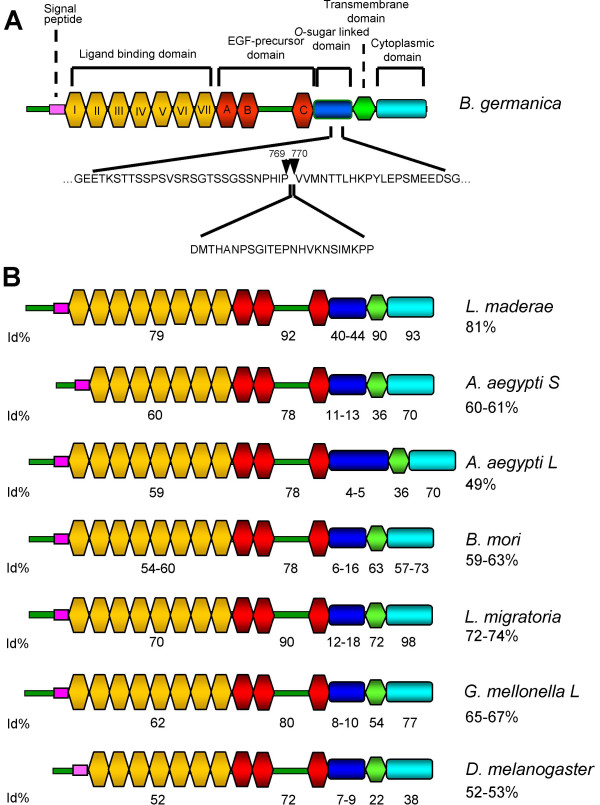
**Organization and sequence comparison of *B. germanica *LpR**. A) BgLpR shows the organization and characteristic domains of a LDL receptor. Two isoforms of BgLpR were found, differing by an insertion/deletion of 24 aa in the *O*-sugar linked domain. B) Comparison of modular domains between different insect LpRs. Identity (%) with respect to *B. germanica *sequence is indicated (overall identity indicated below the species name, and domain identity indicated below the corresponding domain). The complete binomial name of the species can be found in the corresponding section of Methods.

A fragment of *B. germanica *LpR cDNA was cloned by RT-PCR using ovarian and female fat body tissues as templates, and degenerate primers based on conserved motifs of known LpR sequences. Then, 5' and 3' rapid amplification of cDNA ends (RACE) was used to obtain two full-length sequences of 3,120 and 3,195 nucleotides, which encoded each of two proteins of 863 and 887 aa, with predicted molecular masses of 96.6 and 99.2 kDa, respectively. The two cDNAs encoded identical proteins except for an insertion/deletion of 24 amino acids (Fig. 1A). BLAST analysis against databases suggested that the two proteins are LpR orthologues. Therefore, they were called BgLpR-S (*B. germanica *LpR, short form, Accession number GenBank™: AM403064; CAL47126) and BgLpR-L (*B. germanica *LpR, long form, Accession number GenBank™: AM403063; CAL47125).

The putative start codon is preceded by an in-frame stop codon, indicating that these sequences represent full-length ORFs. Conceptual translation showed that both potential proteins (Fig. 1A) have a signal peptide (1–22 aa), followed by a ligand binding domain (LBD) with seven cysteine rich repeats, preceding an epidermal growth factor (EGF) precursor homology domain, with type A, B and C cysteine rich repeats, containing the (Y/F)WXD repeats in number of five. The EGF precursor homology domain is followed by an *O*-linked sugar region; in this region, both BgLpR isoforms differ by the insertion/deletion of 24 aa (Fig. 1A). In the BgLpR-S there is one NX [S/T] motif for the *N*-linked glycosilation of asparagine, whereas in the BgLpR-L there are two of such motifs. Of these two motifs, that which is closer to the 3' end is positioned 15 aa from the transmembrane domain, a distance which suffices for the glycosilation of asparagine [17]. Then, there is a transmembrane domain, rich in aliphatic residues and with a GXXXG motif that breaks the α helix that could be formed by them. Finally, there is a cytoplasmic domain having the clathrin-coated pit internalization motif FDNPVY. Possible sites for co-and post-translational modification other than the *O*-linked sugar region, include 10 *N*-linked glycosylation sites (containing the consensus motif NXS/T) and 53 putative phosphorylation sites (predicted with the NetPhos program [18]) on serine, threonine and tyrosine residues.

The organization and the primary structure of BgLpR were compared with LpRs of other insects (Fig. 1B). BgLpR was most similar to the cockroach *Leucophaea maderae *LpR with 81% of overall identity, and 92% identity when comparing the EGF precursor homology domain. Identity with respect to LpRs of insects belonging to other orders was lower (between 74 and 49% overall identity, and 90–78% when comparing the EGF precursor homology domain) (Fig. 1B).

### Evolutionary relationship of the BgLpR and other lipoprotein receptors

**Figure 2:**
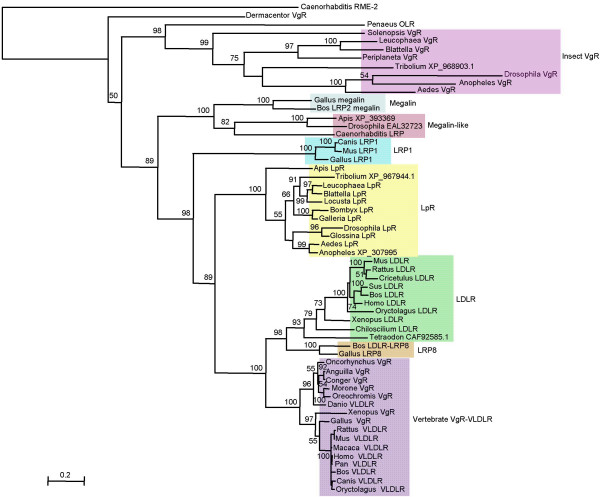
**The *B. germanica *LpR in the context of lipoprotein receptors**. Maximum-likelihood phylogenetic tree showing the position of *B. germanica *LpR with respect to vertebrate and invertebrate lipoprotein receptors. The yolk receptor (RME-2) of the nematode *C. elegans *and the VgR of the tick *D. variabilis *were used as out-groups. Branch lengths are proportional to sequence divergence. The bar represents 0.2 differences per site. Bootstrap values based on 100 replicates are shown on the nodes. The original label for function has been indicated on each species. If there is no functional annotation, then the accession number is indicated. The complete binomial name of the species and the accession number can be found in the corresponding section of Methods.

In order to place the BgLpR in an evolutionary context, we carried out a phylogenetical analysis of lipoprotein receptors available in the literature and databases. Using maximum-likelihood approaches, and the so-called yolk receptor (RME-2) of the nematode *Caenorhabditis elegans *and the vitellogenin receptor (VgR) of the tick *Dermacentor variabilis *as out-groups, we obtained the tree shown in figure 2. BgLpR fall into a strongly supported (100% bootsrap value) cluster of insect LpRs. This cluster is the sister group of another strongly supported cluster (100% boostrap value) formed by vertebrate LDLR + LRP8 + vertebrate VgR-VLDLR. The consistency of the LpR cluster allows to annotate the *Anopheles gambiae *(GenBank™: XP_307995) and *Tribolium castaneum *(GenBank™: XP_967944) sequences as LpRs. Interestingly, insect VgRs appear as a very external cluster, being the sister group of all the remaining lipoprotein receptors. The general good support of the all nodes allows to annotate the sequences of *Apis mellifera *(GenBank™: XP_393369) and *Drosophila pseudoobscura *(GenBank™: EAL32723) as megalin-like, the sequence of *Tetraodon nigroviridis *(GenBank™: CAF92585.1) as LDLR, that of *Danio rerio *(GenBank™: AAH47187, labeled as VLDLR), as fish VgR, and the sequence GenBank™: XP_968903.1 of *T. castaneum*, as insect VgR.

### BgLpR mRNA expression

Expression studies of BgLpR in terms of mRNA were carried out by RT-PCR followed by Southern blotting, using a primer pair that amplifies the two isoforms. The weight of the amplified fragments was in agreement with the expected lengths: 756 bp for BgLpR-S and 828 bp for BgLpR-L. The two isoforms were detected in all tissues analyzed, namely fat body, ovary, brain, midgut, muscle, as well as in embryos (not shown).

**Figure 3:**
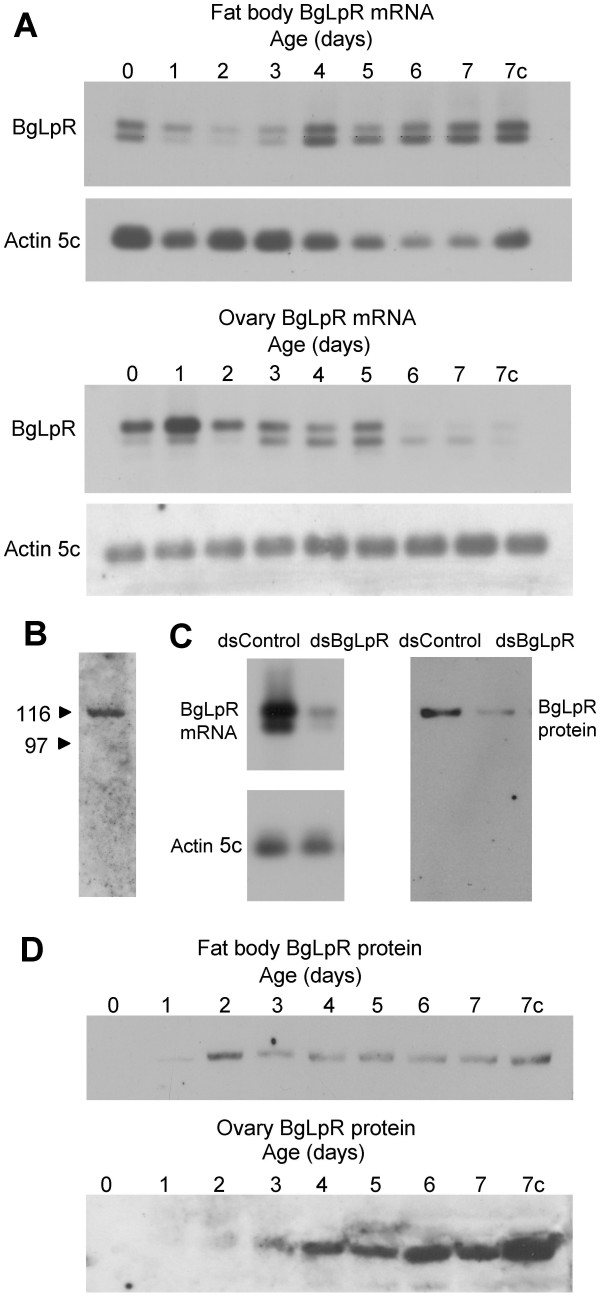
**Expression patterns of BgLpR in the fat body and the ovary of *B. germanica***. The study was carried out in adult females during the 7 days of the first gonadotrophic cycle. 7c means 7-day-old adult females with basal oocytes chorionated. A) BgLpR mRNA expression in fat body and ovary analyzed by RT-PCR/Southern blotting. *B. germanica *actin5C levels were used as a reference. B) The antibody raised against BgLpR recognized a single band in fat body extracts subjected to 7.5% SDS-PAGE under denaturing conditions. C) RNAi experiments to further assess the antibody specificity for BgLpR. Left panel: BgLpR mRNA levels in the fat body of dsControl and dsBgLpR-treated specimens; actin5C levels were used as a reference. Right panel: Western blot analysis using the antibody against BgLpR in the fat body of dsControl and dsBgLpR-treated specimens; the antibody reveals a single band having between 97 and 116 kDa, as in B, corresponding to BgLpR protein. D) Western-blot analysis showing the BgLpR protein pattern in the fat body and the ovary. In the case of fat body, 10 μg of total proteins were loaded in each lane. In that of ovaries, 0.02 ovary equivalents were loaded in each lane. In the later gel, we loaded low amounts of tissue and we exposed the film longer than usual to avoid the "pressing" effect of the huge vitellin band placed just above that of LpR. In films that are exposed even longer, LpR can be also detectable on the days 0, 1 and 2. Blots shown are representative of three replicates.

In the fat body, mRNA expression of the two isoforms during the vitellogenic cycle follow the same pattern, with low levels from day 0 to 3 (previtellogenesis), and higher levels from day 4 to 7 (vitellogenesis) and 7c (choriogenesis) (Fig. 3A). The relatively high intensity of the LpR bands on day 0 is due to loading differences as shown by densitometry data with reference to the actin 5c band. In the ovary, BgLpR-L mRNA levels are high at the beginning of the adult stage but decrease thereafter, being very low on days 6 and 7 and during choriogenesis (7c) (Fig. 3A). Those of BgLpR-S are very low during the first days, increase slightly in mid-vitellogenesis (days 3, 4 and 5) and decrease on days 6 and 7 (Fig. 3A).

### BgLpR protein levels

Firstly, a specific antibody for BgLpR was obtained on the basis of a recombinant LpR fragment corresponding to the EGF precursor domain (from aa 394 to 579), which is exclusive of insect LpRs, and which was used to immunize New Zealand female rabbits. Using crude extracts of fat body and Western blotting, the antibody recognized a single band with a molecular weight between 97 and 116 kDa (Fig. 3B). The suitability of the antibody for BgLpR recognition was further validated with RNAi experiments. Newly emerged adult females were treated with 10 μg of dsRNA targeting BgLpR (dsBgLpR), and fat body tissues were extracted 6 days later and analyzed in terms of mRNA with RT-PCR, and of protein with Western blotting. In dsBgLpR-treated specimens, BgLpR mRNA levels dramatically decreased, as expected, whereas Western blotting showed that the 97–116 kDa band also resulted greatly reduced (Fig. 3C), which indicates that the band correspond to BgLpR protein.

Protein pattern of BgLpR in the fat body (Fig. 3D) is roughly parallel to that of mRNA (Fig. 3A), with low levels at the beginning of gonadotrophic cycle and higher levels from day 2 onwards. In the ovaries, the pattern of BgLpR protein (Fig. 3D) is similar to that in the fat body, with low levels in previtellogenic stages, and higher levels during vitellogenesis and choriogenesis. This pattern contrasts with that of ovarian mRNA (Fig. 3A), which is practically the reverse.

### Effects of JH on BgLpR expression

**Figure 4:**
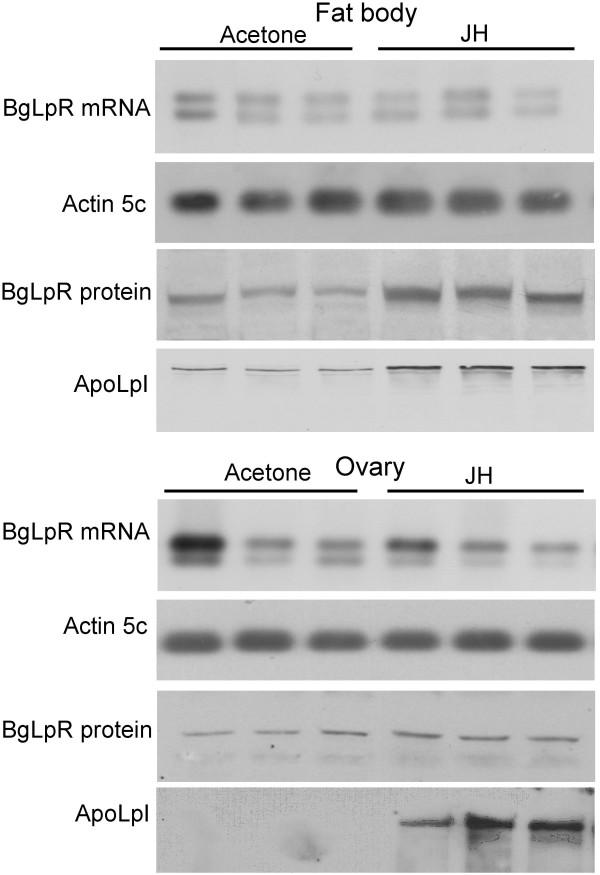
**Effects of juvenile hormone (JH) on BgLpR expression in *B. germanica***. Racemic JH III (10 μg in 1 μl of acetone) was topically applied to newly emerged females of *B. germanica*, and fat body and ovaries were dissected 48 h later. Controls received 1 μl of acetone. BgLpR mRNA levels were studied by semiquantitative RT-PCR/Southern blotting. *B. germanica *actin5C levels were used as a reference. BgLpR protein expression was studied by Western blotting. Effects on apolipophorin I (ApoLpI) were also studied by Western blotting. 10 μg of total proteins (fat body) or 0.2 ovary equivalents (ovary) were loaded in each lane. Blots shown are representative of nine replicates.

BgLpR protein patterns in the fat body and in the ovary are roughly parallel to the profile of JH production [19]. This suggests that JH may play a regulatory role on BgLpR expression. To test this hypothesis, we treated newly emerged females with 10 μg of JH III, and we examined BgLpR levels (mRNA and protein) in the fat body and the ovaries 48 h later. mRNA levels were not affected by the treatment in either of the two tissues (Fig. 4). Conversely, BgLpR protein levels were higher in the specimens treated with JH, especially in the fat body (Fig. 4). Moreover, JH treatment also increased lipophorin levels (estimated in terms of apolipoprotein I) in both tissues (Fig. 4).

### Interfering BgLpR expression by RNAi

**Figure 5:**
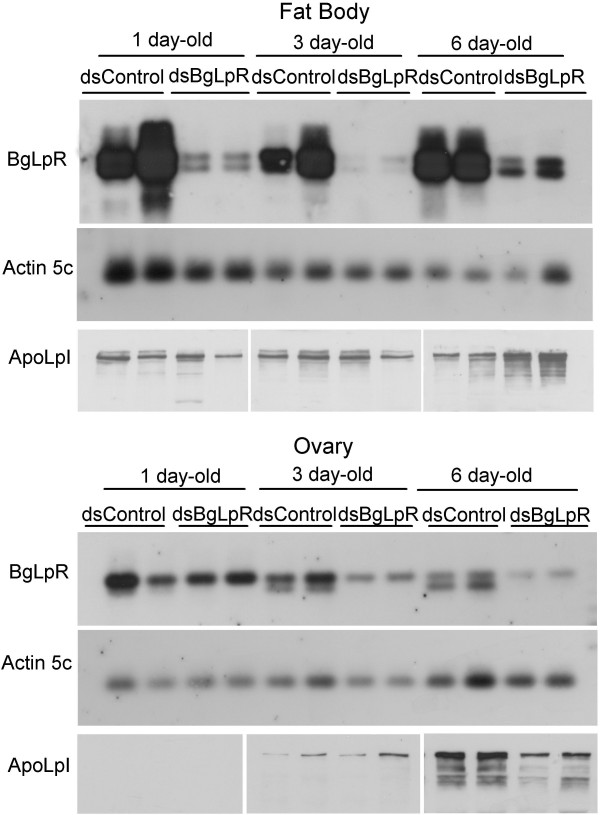
**Interfering BgLpR expression in *B. germanica *by RNAi**. dsBgLpR or dsControl were injected in newly emerged females, and BgLpR mRNA levels were analyzed by RT-PCR/Southern blotting in the fat body and ovaries 1, 3 and 6 days later. *B. germanica *actin5C levels were used as a reference. At the same time intervals, effects on apolipophorin I (ApoLpI) were also studied by Western blotting. 10 μg of total proteins (fat body) or 0.2 ovary equivalents (ovary) were loaded in each lane. Blots shown are representative of six replicates.

In order to assess experimentally the function of BgLpR, we carried out RNAi experiments. Interference of BgLpR should presumably lead to a phenotype with less lipophorin, at least in growing oocytes. Firstly, we aimed at checking whether RNAi would decrease BgLpR mRNA levels. Therefore, 10 μg of dsBgLpR were injected into newly emerged adult females, and mRNA levels were examined 1, 3 and 6 days after the treatment in the fat body and in the ovaries. In the fat body, BgLpR mRNA levels decreased ca. 60% 1 day after the treatment, became very low (ca. 90% of reduction) on day 3, and slightly increased on day 6 (ca. 75% of reduction) (Fig. 5). In the ovary, lowering of BgLpR mRNA levels after RNAi treatment proceeded more slowly. One day after the treatment mRNA levels were similar to controls, but on days 3 and 6, they decreased progressively, reaching ca. 60% reduction on day 6 (Fig. 5).

In control females, lipophorin levels in the fat body are quite constant, comparing days 1, 3 and 6 (Fig. 5). In dsBgLpR-treated specimens, lipophorin levels were not reduced in the fat body, and even increased slightly on day 6, with respect to controls (Fig. 5). In the ovary of control specimens, lipophorin content increased progressively from day 1 to day 6, as described previously [4]. In contrast with the fat body, lipophorin levels were reduced in the ovary 6 days after the treatment with dsBgLpR (Fig. 5), that is when reduction of BgLpR mRNA levels were maximal (Fig. 5).

In spite of having less lipophorin in the ovary, dsBgLpR-treated specimens had no special problems of fertility. In 6-day-old specimens, basal oocyte length was 1.75 ± 0.04 mm (n = 22) in controls, and 1.65 ± 0.03 mm (n = 25) in dsBgLpR-treated specimens (differences not statistically significant, *t*-test: *P *< 0.05). Treated females oviposited and formed the ootheca one day later than controls, but the shape of the ootheca was identical in both groups. The number of nymphs emerging from the ootheca was 40.70 ± 4.02 (n = 7) in controls, and 38.50 ± 2.32 (n = 8) in dsBgLpR-treated specimens (differences not statistically significant, *t*-test: *P *< 0.05). Nymphs emerging from the second ootheca were 33.20 ± 4.85 (n = 5) in controls, and 35.43 ± 1.78 (n = 7) in dsBgLpR-treated specimens (differences not statistically significant, *t*-test: *P *< 0.05).

## Discussion

Two LpR showing the typical organization of a lipoprotein receptor have been cloned and sequenced from the cockroach *B. germanica*. The two sequences (BgLpR-S and BgLpR-L) differ only by an insertion/deletion of 24 aa located in the *O*-linked sugar domain. With the exception of these 24 aa, the two BgLpR sequences are identical, not only in terms of amino acids, but also at level of nucleotide sequence, which suggests that they are splice variants of the same gene. Splice variants differing by a short insertion/deletion in the *O*-linked sugar domain have been also described in the LpR of *A. aegypti *[9,10], *G. mellonella *[11] and *B. mori *[12]. Whereas most of the available insect LpRs have eight cysteine rich repeats within the LBD, the two BgLpR isoforms have only seven. The fat body-specific LpR of *A. aegypti *and the single LpR reported in *D. melanogaster *[10,20,21] have also seven cysteine rich repeats, and this has led to propose that the later could be fat body-specific [10]. However, the case of *B. germanica *shows that the occurrence of only seven repeats do not define fat body specificity, because BgLpR-S and BgLpR-L are expressed irrespectively in the fat body and in the ovary (as well as in brain, midgut, muscle and embryo tissues).

Maximum-likelihood phylogenetical analyses show that LpRs form a monophyletic group, and that LpRs and the cluster formed by vertebrate LDLR + LPR8 + VgR + VLDLR are sister groups. Rodenburg and coworkers [20] proposed that LDLR proteins can be classified on the basis of the amino acid sequence of their C-terminal domains. The analysis reported by these authors is represented by an unrooted tree, but the clusters obtained are similar to those described herein. The main difference between these two studies is that we used more sequences (including insect VgRs), and that our rooted tree allows a more visual reconstruction of the molecular evolution of the sequences involved.

In the fat body, mRNA levels of both BgLpR isoforms increase with time during the first vitellogenic cycle, whereas they decrease in the ovary. This suggests that the mechanisms regulating BgLpR expression differ in these two organs. Conversely, BgLpR protein levels increase in parallel to vitellogenesis either in the fat body as well as in the ovary [22]. In the ovary, the BgLpR pattern also resembles that of vitellogenin receptor (BgVgR) expression [23]. Using Western blotting, the antibody developed in the present work recognized a single band with a molecular weight between 97 and 116 kDa, which is slightly higher than that predicted for the heavier BgLpR-L isoform (96.6 kDa), a difference that might be due to posttranslational modifications. RNAi of BgLpR resulted in a clear decrease of this band, which indicates that it corresponds to BgLpR. The fact that only a single band is revealed by the antibody suggests that only one of the isoforms is translated, as occurs in the LpR of *G. mellonella*, were only the splice variant containing the full *O*-linked sugar domain is translated into the receptor protein [21]. We cannot exclude the possibility that the isoform not present in the fat body and the ovary is translated and plays a tissue-specific role in other tissues or stages were BgLpR is transcribed (brain, midgut, muscle, embryos).

Treatment with JH increased the levels of BgLpR protein, especially in the fat body, but not those of BgLpR mRNA. Posttranscriptional effect of JH upon BgLpR could be related to mRNA stability, mRNA transport or the own translation rate. Antecedents of posttranscriptional effects of JH on protein expression have been described in *A. aegypti*. In this mosquito, JH enhances the synthesis of 8FTZ-F1, an orphan nuclear receptor involved in the acquisition of competence to 20-hydroxyecdysone by the fat body to produce vitellogenin [24]. In *G. mellonella*, production of LpR protein is also hormonally enhanced, but by 20-hydroxyecdysone [21]. Our experiments also showed that JH-treated specimens had higher levels of lipophorin, estimated in terms of apolipophorin I (ApoLpI). However, this likely derive from the increased levels of BgLpR, given that JH do not seem to regulate lipophorin levels directly [4,5], although in the ovary, higher levels of ApoLpI can also be due to the enhanced basal oocyte growth provoked by the treatment with JH.

In order to demonstrate the function of BgLpR as lipophorin receptor, we interfered BgLpR expression by systemic RNAi, studying the effects in the ovary and the fat body. In the ovary, BgLpR mRNA levels decreased progressively after the treatment with dsBgLpR, and on day 6 they were clearly lower than in controls (60%). On the same day, lipophorin levels in the ovary of dsBgLpR-treated females were much lower than in controls, as expected. In controls, and as previously described [4], lipophorin contents in the ovary increase in parallel to vitellogenesis. The situation in the fat body is more complex, because this organ not only internalizes but also synthesizes lipophorins. Three days after the treatment with dsBgLpR, BgLpR mRNA levels practically vanished in the fat body, but lipophorin levels were almost indistinguishable from controls. Three days later, levels of BgLpR mRNA and lipophorin contents increased. This double increase suggests that knockdown effects began to disappear. Expression recovery of the target gene after dsRNA treatment, which has been described in other systems, like in the eye color gene *vermilion *in the locust *Schistocerca gregaria *[25], suggests that RNAi must be used carefully when suspecting that the resulting phenotype can be transient.

## Conclusion

RNAi experiments demonstrate that the BgLpR cloned and sequenced in *B. germanica *is a functional lipophorin receptor. Expression studies and experiments with JH, suggest that expression of BgLpR is posttranscriptionally regulated by this hormone. Phylogenetical analysis indicates that insect LpRs form a well supported monophyletic group. Our functional studies on a cockroach based on RNAi, together with functional studies based on biochemical approaches on locusts and mosquitoes, strongly supports the notion that the LpR group is formed by functionally active lipophorin receptors.

## Methods

### Insects

Specimens of *B. germanica *were obtained from a colony reared in the dark at 30 ± 1°C and 60–70% r.h. Freshly ecdysed adult females were selected and used at appropriate ages. All dissections and tissue sampling were carried out on carbon dioxide-anaesthetized specimens.

### Cloning and sequencing

Degenerate primers based on conserved sequences of the EGF precursor homology domain of the LpR of *A. aegypti *and *L. migratoria *were used to obtain a *B. germanica *homologue cDNA fragment by PCR amplification using cDNA template generated by reverse transcription from polyA^+ ^RNA from 3-day-old adult ovaries. The amplified fragment (930 bp) was subcloned into pSTBlue™-1 vector (Novagen Madrid, Spain) and sequenced. To complete the BgLpR cDNA sequences, 5'- and 3'-RACE were applied to polyA^+ ^RNA extracted from ovaries and fat body using a 5'- and 3'-RACE system Version 2.0 (Invitrogen, Paisley, UK), according to the manufacturer's instructions. The PCR products were analyzed by agarose gel electrophoresis, cloned into pSTBlue™-1 vector and sequenced. For 5'-RACE, reverse primers were as follows: LpR-R1 (position 1294–1314), 5'-AGCGAACAGAAGAGAGGCATG-3', and nested LpR-R2 (position 1271–1291), 5'-CTTCCATGGCTTTACATCTCG-3'. For 3'-RACE, forward primer was as follows: LpR-F1 (position 1601–1622) 5'-TCGAGGGCAACATGAGGAAGAT-3'.

### Sequence comparisons and phylogenetical analysis

Sequences of lipoprotein receptors *sensu lato *were retrieved from GenBank protein database. These included the insect LpRs of *Galleria mellonella *(GenBank™: ABF20542), *Bombyx mori *(GenBank™: BAE71406.1), *Aedes aegypti *(GenBank™: AAQ16410 and GenBank™: AAK72954), *Drosophila melanogaster *(GenBank™: NP_733119.1), *Glossina morsitans *(GenBank™: ABC48942.1), *Leucophaea maderae *(GenBank™: BAE00010), *Locusta migratoria *(GenBank™: CAA03855) and *Apis mellifera *(GenBank™: XP_395858.2); the insect VgRs of *Drosophila melanogaster *(GenBank™: AAB60217), *Anopheles gambiae *(GenBank™: EAA06264), *Aedes aegypti *(GenBank™: AAK15810), *Solenopsis invicta *(GenBank™: AAP92450), *Periplaneta americana *(GenBank™: BAC02725), *Leucophaea maderae *(GenBank™: BAE93218.1) and *Blattella germanica *(GenBank™: CAJ19121); the ovarian lipoprotein receptor (OLR) of the crustacean *Penaeus semisulcatus *(GenBank™: AAL79675.1); the VgR of the tick *Dermacentor variabilis *(GenBank™: AAZ31260.3); the yolk receptor RME-2 of the nematode *Caenorhabditis elegans *(GenBank™: AAD56241.1); and the VgR of the vertebrates *Anguilla japonica *(GenBank™: BAB64337.1), *Conger myriaster *(GenBank™: BAB64338), *Oncorhynchus mykiss *(GenBank™: CAD10640.1), *Oreochromis aureus *(GenBank™: AA027569.1), *Morone americana *(GenBank™: AA092396.1), *Xenopus laevis *(GenBank™: AAH70552) and *Gallus gallus *(GenBank™: NP_990560). We also included the very low density lipoprotein receptor (VLDLR) of *Danio rerio *(GenBank™: AAH47187), *Canis familiaris *(GenBank™: XP_533538.2), *Oryctolagus cuniculus *(GenBank™: BAA01874), *Rattus norvegicus *(GenBank™: NP_037287.1), *Mus musculus *(GenBank™: AAH13622.1), *Bos taurus *(GenBank™: NP_776914.1), *Macaca mulatta *(GenBank™: AAR83314.1), *Pan troglodytes *(GenBank™: XP_520460.1) and *Homo sapiens *(GenBank™: NP_003374.3); the low density lipoprotein receptor (LDLR) of *Chiloscyllium plagiosum *(GenBank™: AAB42184.1), *Xenopus laevis *(GenBank™: Q99088), *Cricetulus griseus *(GenBank™: P35950), *Mus musculus *(GenBank™: CAA45759.1), *Rattus norvegicus *(GenBank™: NP_ 786938.1), *Oryctolagus cuniculus *(GenBank™: P20063), *Sus scrofa *(GenBank™: AAC17444.1), *Bos taurus *(GenBank™: XP_874020.1) and *Homo sapiens *(GenBank™: NP_000518.1); the lipoprotein receptor-related protein (LRP) 8 of *Bos taurus *(GenBank™: XP_870184.1) and *Gallus gallus *(GenBank™: CAA65729.1); the LRP1 of *Mus musculus *(GenBank™: NP_032538.1), *Canis familiaris *(GenBank™: XP_538245.2) and *Gallus gallus *(GenBank™: NP_990573.1); the LRP of *Caenorhabditis elegans *(GenBank™: Q04833); and the megalins (= LRP2) of *Bos taurus *(GenBank™: XP_ 592673.2) and *Gallus gallus *(GenBank™: XP_422014.1). In addition, we also included the sequences of putative lipoprotein receptors of *Drosophila pseudoobscura *(GenBank™: EAL32723), *Tribolium castaneum *(GenBank™: XP_967944 and GenBank™: XP_968903.1), *Anopheles gambiae *(GenBank™: XP_307995), *Apis. mellifera *(GenBank™: XP_393369) and *Tetraodon nigroviridis *(GenBank™: CAF92585.1), which were not annotated. Protein sequences were aligned using clustal X [26]. Poorly aligned positions and divergent regions were eliminated by using Gblocks 0.91b [27]. The resulting alignment was analyzed by the PHYML program [28] based on the maximum-likelihood principle with the amino acid substitution model. Four substitution rate categories with a gamma shape parameter of 1.444 were used. The data was bootstrapped for 100 replicates using PHYML.

### RT-PCR/Southern blot analyses

Temporal profiles of transcript variation for the two BgLpR isoforms were carried out on ovary and fat body tissues using RT-PCR followed by Southern blotting with specific probes. For each determination, ovary or fat body total RNA from pools of four to six individuals were isolated using the Gen Elute Mammalian Total RNA kit (Sigma, Madrid, Spain). An amount of 400 ng of each RNA extraction was DNAse treated (Promega Biotech Iberica. Madrid, Spain) and reverse transcribed with Superscript II reverse transcriptase (Invitrogen) and random hexamers (Promega). PCR amplification of the two BgLpR isoforms was carried out simultaneously in a single reaction containing the following primers: forward 5'-TCGCCCGATGCCTTGCAACA-3' and reverse 5'-TACCACTTGGCGCATTCTATG-3'. As a reference, the same cDNAs were subjected to RT-PCR/Southern blotting with a primer pair specific to *B. germanica *actin5C [29]. cDNA probes for Southern blot analyses were generated by PCR with the same primer pairs, using plasmid DNA containing the corresponding cDNA clones as template. The probes were labeled with fluorescein with the Gene Images random prime-labeling module from GE Healthcare (Barcelona, Spain). RT-PCR followed by Southern blotting of total RNA without reverse transcription was carried out in parallel to assess that there was not genomic contamination.

### BgLpR antibody

A 556 bp DNA fragment (from aa 394 to aa 579), corresponding to EGF precursor homology domain of the BgLpR (a domain which is exclusive of insect LpRs) was chosen to produce a BgLpR recombinant fragment to generate the corresponding polyclonal antibody. The restriction enzyme sites *EcoRI *and *XhoI *were added to the specific primers used to amplify the fragment. The PCR amplified fragment was cloned into pSTBlue™-1 vector and sequenced. Then, it was purified by agarose gel electrophoresis, digested with *EcoRI *and *XhoI *and directionally ligated into pET28a(+) expression vector from Novagen. *E. coli *BL21(DE3)plysS competent cells were used for plasmid transformation. The transformed bacteria were selected by screening the colonies on media containing 30 μg/ml of kanamycin. Colonies were further analyzed by restriction enzyme digestion and PCR. Bacteria were grown until the OD_600nm _reached 0.6 and were induced with 0.8 mM of IPTG for 3 h. The expressed protein was purified using Ni-NTA from Qiagen (Barcelona, Spain) column according to the manufacturer's instructions. The purified recombinant BgLpR fragment was quantified [30], and quality was tested by SDS-PAGE 13% stained with Coomassie blue. The eluted 21 kDa band was homogenized in Ringer solution, emulsified with complete Freund's adjuvant, and used to boost New Zealand female rabbits.

### Characterization of the antibody and immunoblot analyses

For immunoblot analyses, ovaries and fat bodies were dissected under Ringer solution, frozen with liquid N_2 _and preserved at -80°C until use. For protein extraction, tissues were homogenized in 100 μl of a buffer composed of 100 mM sucrose, 40 mM K_2_HPO_4 _pH 7.2, 30 mM EDTA, 50 mM KCl, 0.25% (v/v) Triton X-100, 10 mM DTT and 0.5 mM proteases inhibitor cocktail (Roche diagnostics. Barcelona, Spain). After measuring the protein contents of homogenates [30], suramine (Sigma) (5 mM) was added to inhibit the binding of lipophorin to its receptor, and incubation was left to proceed during 1 h. Homogenates were analyzed in 7.5% SDS-PAGE gels under denaturing conditions and loading the same protein quantity in both ovary and fat body. Gels were transferred to a nitrocellulose membrane (Schleicher and Schuell, Dassel-Germany), incubated with BgLpR antibody (see above) (serum was used at 1:8,000) for 2 h, or with *B. germanica *ApoLpI antibody (a gift from C. Schal) (1:16,000) for 1 h to detect lipophorin in terms ApoLpI [4], and then were processed with SuperSignal West Femto Maximum Sensitivity Substrate (Pierce, Rockford, IL), following the manufacturer's instructions.

### Juvenile hormone experiments

Newly emerged adult females were topically treated with 10 μg of racemic JH III (Sigma) in 1 μl acetone. Controls received the same volume of acetone. Ovaries and fat body were explanted 48 h after the treatment, in order to examine the effects of JH on BgLpR mRNA and protein.

### RNAi experiments

To obtain a dsRNA targeted to BgLpR mRNA (dsBgLpR), a 556 bp fragment corresponding to EGF precursor homology domain of BgLpR (from aa 398 to aa 581) was amplified by PCR and subcloned into the pSTBlue™-1 vector. As control dsRNA, we used a non-coding sequence from the pSTBlue-1 vector (dsControl)[31]. Single stranded sense and antisense RNAs were obtained by transcription in vitro using either SP6 or T7 RNA polymerases from the respective plasmids, and resuspended in water. To generate the dsRNAs, equimolar amounts of sense and antisense RNAs were mixed, heated at 95°C for 10 min, cooled slowly to room temperature and stored at -20°C until use. Formation of dsRNA was confirmed by running 1 μl of the reaction products in 1% agarose gel. The obtained dsBgLpR was resuspended in diethyl pyrocarbonate-treated water and diluted in Ringer saline at a concentration of 5 μg/μl. In all experiments, a single dose of 10 μg was injected into the abdomen of newly emerged females, and the effects were examined at different times during the first and the second reproductive cycle.

### Statistics

Data are expressed as mean ± standard error of the mean (SEM). Differences between groups were tested by Student's *t*-test.

## Abbreviations

JH, juvenile hormone; LpR, lipophorin receptor; ApoLpI, Apolipophorin I; UTR, untranslated region; EGF, epidermal growth factor; LRP, lipoprotein receptor-related protein; LDLR, low density lipoprotein receptor; RACE, rapid amplification of cDNA ends; VLDLR, very low density lipoprotein receptor; VgR vitellogenin receptor.

## Authors' contributions

LC carried out most of the experiments. XB participated in the coordination, contributed to phylogenetical studies and wrote the last version of the manuscript. MDP conceived the study, contributed to performing experiments and phylogenetical analysis, and drafted the manuscript. All authors read and approved the final manuscript.
